# Clinical Presentation and Platelet Profile of Dengue Fever: A Retrospective Study

**DOI:** 10.7759/cureus.28626

**Published:** 2022-08-31

**Authors:** J. Vijay, N. Anuradha, Viknesh P Anbalagan

**Affiliations:** 1 Department of General Medicine, Sree Balaji Medical College and Hospital, Chennai, IND

**Keywords:** ascites, pleural effusion, platelet count, myalgia, dengue fever

## Abstract

Background: Dengue fever (DF) is a mosquito-borne viral illness carried worldwide by *Aedes aegypti* and *Aedes albopictus* mosquitoes. The aim of the present study was to observe the different clinical presentations of dengue fever and the platelet profile analysis in DF patients.

Methods: This retrospective study was performed on 130 diagnosed patients with dengue fever, aged over 14 years. Data collection included patient age, gender, clinical manifestations, hematocrit, platelet count, and evidence of plasma leakage.

Results: Most of the patients belong to the 21-30 year age group, with a greater number of males compared to females. The common presentation of dengue fever was fever and myalgia, observed in 100% and 95.3% of the patients, respectively. A platelet count of less than 1,00,000 was observed in 77% of patients, whereas decreased total leukocyte count (TLC) and hematocrit were observed in 52.3% and 40% of patients, respectively. About 46.15% of patients had bradycardia on examination. Pleural effusion and ascites were found in 20.7% and 15.3% of patients, respectively.

Conclusion: Patients presenting with fever, hemorrhagic symptoms, or signs of plasma leakage should be promptly suspected, timely diagnosed and managed on the grounds of dengue fever.

## Introduction

Dengue fever (DF) is an acute, self-limiting systemic viral illness caused by the dengue virus (Flaviviridae), spread globally by the mosquitoes *Aedes aegypti* and *Aedes albopictus* [1]. The World Health Organization (WHO) listed DF as one of the top ten global health risks. The dengue virus infects an estimated 390 million people each year, with 96 million showing clinical symptoms. In the previous decade, the number of cases in Southeast Asia has grown [2]. In tropical and sub-tropical nations, DF is a public health issue. Epidemics are growing increasingly common in India, burdening the public health system's limited resources. In India, the epidemic of dengue patients has increased in the past. Dengue epidemics in India are cyclical and spread geographically into rural regions, and cycle all sorts of serotypes in the population [3]. Specific clinical criteria identify dengue cases; however, they can appear with various symptoms. DF is a mysterious disease, including the virus-vector and host-virus relationships and a wide range of clinical manifestations [4].

Dengue infection manifests itself in a variety of ways, from mild febrile fever (DF) to severe hemorrhagic diseases like dengue hemorrhagic fever (DHF) and dengue shock syndrome (DSS) [5]. The deadliest variants of this disease, DHF and DSS, have been documented in India from Delhi, Calcutta, and Chennai [6]. Over the decade following the first epidemic, there has been a temporal change in the prevalence of various clinical symptoms. The change in the clinical presentation was thought to be caused by shifting serotypes (DEN-1, 2, 3, and 4) throughout outbreaks and re-infection. In addition, detailed serotype data for each occurrence is yet unavailable [7].

Antipyretics and pain relievers treat DF asymptomatically to relieve muscle and bone discomfort. Severe instances may necessitate hospitalization as well as enough hydration. The febrile phase of DF is marked by a high fever, headache, myalgia, body soreness, vomiting, joint pain, temporary rash, and modest bleeding symptoms such as petechiae, ecchymosis at pressure sites, and venipuncture bleeding [8]. The patient's risk of progressing to severe dengue (SD) is increased in the following critical phase, defined by plasma leakage that can lead to shock and fluid buildup (ascites or pleural effusion) with severe bleeding without respiratory difficulty and severe organ damage [9].

Acute liver failure, encephalopathy with convulsions, renal dysfunction, and lower gastrointestinal hemorrhage are all examples of unusual presentations. The clinico-epidemiologic characteristics of dengue infection have already been studied in several publications [10]. Patients with dengue fever who presented to the outpatient or emergency departments of a tertiary care hospital in an urban environment were assessed for their clinical and hematological profiles. The aim of the present study was to observe the different clinical presentations of dengue fever and the platelet profile analysis in DF patients.

## Materials and methods

The present observational study was conducted at a tertiary care hospital over 24 months during the dengue fever season between 2019 and 2021. All patients presenting to the outpatient department with complaints of fever and clinical features of dengue with a positive test (dengue NS1) were included in this study. The study was conducted in accordance with the ethical principles following approval from the Medical Review and Ethical Committee (Registration No. ECR/719/Inst/TN/2015/RR-21). Written informed consent was obtained from every volunteer before clinical trial participation. The study includes all the patients meeting the inclusion criteria who gave consent from the dengue ward of the hospital; male and female patients above 14 years of age with bleeding manifestations and thrombocytopenia with platelet count (less than or equal to 100,000/μL). Patients with other viral or bacterial infections after a routine lab test and those who refused to participate in the survey were excluded from the study.

Data collection

At the time of presentation, the following information was collected: age, gender, clinical presentation, duration of fever, myalgia, joint pain, vomiting, rash, bleeding, hepatomegaly, headache, and shock, along with plasma leakage evidence such as ascites, pleural effusion, presence of petechiae, positive tourniquet test, other bleeding manifestations, hematocrit, and platelet count. Based on the presence of clinical symptoms, patients were classified as having dengue fever without warning signals (DF), dengue fever with warning signs (DFWS), or severe dengue (SD). The data were tabulated and presented as numbers (percentages).

## Results

All the patients were successfully enrolled in the study. Most of the patients were in the age group of 21-30 years (60), followed by 31-40 years (30), 15-20 years (22), 41-50 years (11), and 51-60 years (7). Out of all patients, 80 (62%) patients were males. The severity of dengue increased in 22% of patients with DHF and 6% with DSS. Male patients predominated in the DHF and DSS categories (20 and 6%). Relatively lower female patients were observed in groups DHF (8%) and DSS (2%) (Table 1). Among the patients, all were diagnosed with fever (100%), myalgia (95.3%), headache (79.20%), as well as joint pain (76.90%), which were observed in higher frequencies. Shock (93.80%), bleeding (78.40%), and hepatomegaly (75%) were not observed in most of the patients. Hess was observed only in 26.1% of patients.

**Table 1:** Gender-specific clinical syndrome of dengue patients. DF: dengue fever, DHF: dengue hemorrhagic fever, DSS: dengue shock syndrome.

Variables	DF	DHF	DSS
Sex	Number (%)	Number (%)	Number (%)
Female	40 (43)	8 (29)	2 (25)
Male	54 (57)	20 (71)	6 (75)
Total	94 (100)	28 (100)	8 (100)

Among the patients, all were diagnosed with fever (100%), myalgia (95.3%), headache (79.20%), as well as joint pain (76.90%), which was observed in higher frequencies. Shock (93.80%), bleeding (78.40%), and hepatomegaly (75%) were not observed in most of the patients. Hess was observed only in 26.1% of patients (Table 2).

**Table 2:** Clinical features of patients with dengue fever. ABD: Abdomen.

Variables	Symptoms present	Symptoms absent
	Number (%)	Number (%)
Fever	130 (100)	0 (0)
Myalgia	124 (95.30)	6 (4.60)
Joint pain	100 (76.90)	30 (23.00)
Vomiting	77 (59.20)	53 (40.70)
Pain ABD	74 (56.90)	56 (43.00)
Rash	59 (45.30)	71 (54.60)
Bleeding	28 (21.50)	102 (78.40)
Headache	103 (79.20)	27 (20.70)
Hepatomegaly	33 (25)	97 (75)
Shock	8 (6.10)	122 (93.80)

On analyzing the hematological parameters, 77% of patients had a platelet count of less than 1,00,000 cu. mm. (Table 3). The total leukocyte count (TLC) was less than 4000 and more than 11,000 in 51% and 3%, respectively. However, 46% of patients had normal TLC. The hematocrit level was less than 40 in 68 (52.3%), between 40-45 in 37 (28.4%), and more than 45 in 25 (19.2%) patients (Table 3). In 60 (46.15%) patients, bradycardia was observed (Table 3).

**Table 3:** Laboratory hematological parameters.

Parameters		Normal reference value	Number (%)
Platelet count	<100,000	1,00,000-2,50,000 per microliter	100 (77)
	>100,000		30 (23)
	Total		130 (100)
Total leukocyte count	<4000 cu.mm	4000-11,000 cu.mm	66 (51)
	>11,000 cu.mm		5 (3)
	4000-11,000 cu.mm		59 (46)
	Total		130 (100)
Hematocrit	Below 40	35.5% to 44.9%	68 (52.3)
	40–45		37 (28.4)
	Above 45		25 (19.2)
	Total		130 (100)
Bradycardia	Present	60-90 beats per minute	60 (46.15)
	Absent		70 (53.8)

Among the evidence of plasma leakage, 20.7% was reported with pleural effusion, followed by ascites (15.3%), pedal edema (9.2%), and shock (6.15%) (Table 4).

**Table 4:** Bleeding manifestation in dengue patients.

Serial number	Evidence of plasma leakage	Number (%)
1.	Pedal edema	12 (9.2)
2.	Ascites	20 (15.3)
3.	Pleural effusion	27 (20.7)
4.	Shock	8 (6.15)

## Discussion

The present study was conducted to find out various clinical and laboratory parameters of dengue patients attending our hospital. The study aims to have a detailed clinico-hematologic profile of dengue disease so that prompt management of needy patients can be done. The study was conducted on 130 seropositive patients with confirmed dengue fever. Mostly, the disease was observed among young males between 21 and 30 years of age. The most commonly observed syndrome was DF compared to DHF and DSS. However, the most common manifestations were fever and myalgia, observed in 100% and 95.3% of the patients, respectively. On laboratory analysis of hematological parameters, the study revealed that 77% of patients had thrombocytopenia and 51% suffered from leukopenia. Moreover, a hematocrit of less than 40 was observed among 60 patients (52.3%). Bradycardia was found in 60 (46.15%) of the patients. Pleural effusion and ascites were recorded as the most common plasma leakage symptoms.

The present study found that the DF type of dengue is more common in men in their second decade of life. Several investigations have found comparable infection dominance in male patients. The studies relate the commonness to the relatively higher exposure rates of the virus in men [11,12]. Dengue fever is categorized as DF, DHF, or DSS, depending on the severity of the clinical manifestations [11]. These results were relatable to those published earlier that recorded a higher incidence of DF (65.2%) compared to DHF (34%) and DSS (0.79%) among 756 dengue patients studied [13].

Patients with classic dengue fever, arthralgia, myalgia, retro-orbital discomfort, rash, and hemorrhagic signs with or without shock frequently appear with a triad of symptoms. In recent years, respiratory symptoms, gastrointestinal problems, a low platelet count, and abnormal liver function tests have all been reported as signs of dengue fever. Over the decade following the first epidemic, there has been a temporal change in the prevalence of various clinical symptoms [14]. A likely triad of manifestations was observed in our study, with fever and myalgia dominating with other less common indexes: headache, joint pain, vomiting, abdominal pain, rash, hepatomegaly, bleeding, and shock. Fever as a significant index was reported in outbreaks in 2010 and 2018 [14].

The current study observed a lower Hess value (26.1%) that was not relatable to the confirmed dengue cases. In a parallel analysis, a very low percent tested positive for Hess among a large proportion of patients confirmed with dengue [14]. This demonstrates that the tourniquet test is specific but not sensitive for diagnosing dengue fever. For other tropical illnesses, the tourniquet test is not included in the case definition [15]. Due to a low platelet count and increased capillary permeability, hemorrhagic manifestation is one of the consequences of DF [16]. This was evident in most of our patients; 77% recorded a lower platelet count. On the present duty, we couldn't find any patients with bleeding symptoms and a positive tourniquet test. Most of the patients in our study had neutropenia and our findings correlate with the study of Singh et al., who also observed similar low TLC counts [17]. An inconsistency in the TLC of dengue patients was also noted, similar to our study [18]. This research also implicates that virus-induced destruction or suppression of myeloid progenitor cells may cause leukopenia in dengue fever. Bradycardia, one of the major dengue manifestations, was found to occur in 46.15% of patients. A similar relatively lower bradycardia occurrence was reported [19]. Transitions to plasma leakage, which resulted in respiratory distress syndrome and organ failure, were seen more frequently and were thought to predict increased case fatality among dengue patients [20]. Pleural effusion and ascites were the most common bleeding manifestations as per our study. A likely occurrence of ascites, as well as pleural effusion in dengue patients, was reported [21].

Limitations

This study used a limited sample size since it was conducted in a single outpatient clinic. However, because of the low rate of clinics receiving only people from the neighborhood, this small sample was kept for such a long period. In addition, there was no information on whether the infection was primary or secondary and the dengue serotype. Cross-sectional research involving many centers and tertiary hospitals, with a bigger sample size and the entire general population, might yield more helpful conclusions.

## Conclusions

Dengue fever is a serious endemic disease, particularly in developing countries. The present study address common clinical and hematological manifestations of dengue fever together with other syndromes. Fever and myalgia were found to be the most common symptoms in dengue-affected patients. Furthermore, the study emphasizes that by knowing the various clinico-hematological manifestations of dengue fever, timely measures of management and treatment can be undertaken.
